# Values of tumor volume on magnetic resonance imaging for a surgical approach to endometrial cancer

**DOI:** 10.1002/cam4.6384

**Published:** 2023-08-21

**Authors:** Elga López‐González, Alberto Rodríguez‐Jiménez, José Antonio Rojas‐Luna, Cinta Daza‐Manzano, Juan Gómez‐Salgado

**Affiliations:** ^1^ Gynecological Oncology Unit, Department of Obstetrics and Gynecology Hospital Universitario Juan Ramón Jiménez Huelva Spain; ^2^ Department of Radiology Hospital Universitario Juan Ramón Jiménez Huelva Spain; ^3^ Department of Sociology, Social Work and Public Health, Faculty of Labor Sciences University of Huelva Huelva Spain; ^4^ Safety and Health Postgraduate Program Universidad Espíritu Santo Guayaquil Ecuador

**Keywords:** endometrial cancer, lymph node metastases, lymphadenectomy, MRI, tumor volume

## Abstract

**Objective:**

To analyze the relationship between tumor volume in Endometrial Cancer (EC) on Magnetic Resonance Imaging (MRI) and lymph node metastasis to establish which patients benefit from omitting the lymphadenectomy.

**Methods:**

A retrospective observational study with 194 patients with EC identified between 2016 and 2021 at the Juan Ramón Jiménez University Hospital, Huelva (Spain) was carried out. Preoperative MRI of 127 patients was assessed. The tumor volume was analyzed on MRI by the ellipsoid formula and another alternative method with a manual ROI in different sections. Risk factors for node metastases were analyzed to understand its relationship and to identify an optimum criterion for the tailored surgery.

**Results:**

Univariate analysis showed risk factors for lymph node metastases were histological grade (*p* = 0.001), tumor with a volume greater than >25 cm^3^ (*p* < 0.001), lymphovascular space invaded (*p* = 0.007), and preoperative Ca 125 serum >28 (*p* < 0.001). Multivariate analysis indicated that tumor volume index >25 cm^3^ was an independent risk factor for lymph node metastases. The patients without significant proposed risk factors (volume index >25 cm^3^ [OR = 0.64], Ca 125 > 28 [OR = 0.32], and high histological grade [OR = 2.6]) did not present lymph node metastases, independent of myometrial invasion.

**Conclusions:**

Lymphadenectomy can be omitted in patients with Endometrioid carcinoma that do not have any of the following risk factors: high‐grade tumor, elevated Ca 125 (>28), and tumor volume on MRI greater than 25 cm^3^. Tumor volume might predict the state of lymph nodes in EC and it could give information regarding surgical management.

## 1 INTRODUCTION

Endometrial cancer (EC) is the most common gynecological cancer in developed countries and the incidence rate has been increasing over the past decades, according to the Globocan 2020 report with data from the International Agency for Research on Cancer (IARC).^1^, ^2^

Lymph node metastasis (LNM) is an important risk factor for survival in EC. The therapeutic effects of pelvic and para‐aortic lymph node removal is a matter of great debate.^3^, ^4^ In this regard, there is no consensus on whether systematic lymphadenectomy maximizes the therapeutic effect of this procedure and reduces the invasiveness of the surgery. For this reason, classifying patients according to their stage of risk for surgical planning is needed. The International Federation of Gynecology and Obstetrics (FIGO) stage is an evidence‐based document that guides toward prognostic factors in EC and its surgical planning.^5^ Low‐risk^6^ EC is identified with endometroid/grade 1–2 histological type, myoinvasion <50% and no imaging of cervical invasion, or node or pelvic metastases. These patients do not show decreasing risk of death or disease recurrence with the lymphadenectomy. Surgical treatment is planned based on the preoperative assessment of the histological subtype and depending on the grade and depth of myometrial invasion, as observed in the uterine imaging.^7^

The pelvic magnetic resonance imaging (MRI) is the method of choice for preoperative staging.^8^, ^9^, ^10^ The MRI allows the introduction of other parameters, which optimize the presurgical study versus the ultrasound. However, conventional pelvic MRI has reported limitations in detecting the staging parameters and could result in overestimation or underestimation of the depth of myometrial invasion,^11^, ^12^ and, therefore, the advantage of assessing the tumor volume preoperatively is lost.^13^, ^14^

It is already known that tumor size is an important prognostic factor in EC, existing a strong correlation between tumor size higher than 2 cm and the risk of nodal disease and worse overall survival, even in patients in an early stage and low‐grade EC.^15^, ^16^, ^17^ Although the tumor size is a determinant prognostic factor in the adjuvant treatment after the surgery, it is not standardized as an additional parameter in the preoperative study when it comes to assessing the surgical decision.

The main objective of this research was to explore the relationship between the tumor volume in the EC in the MRI and the lymph node metastasis.

## 2 MATERIALS AND METHODS

### 2.1 Study design

An observational retrospective study of a prospective database was conducted. 194 women diagnosed with EC between January 2016 and December 2021 at the Juan Ramón Jiménez Hospital in Huelva (Spain) were included.

127 patients were studied preoperatively using magnetic resonance imaging. All patients were treated according to current guidelines. For the analytical study, only these 127 patients were included, diagnosed with low‐intermediate‐high‐risk endometrioid carcinoma with preoperative MRI and staging surgery performed. The study was approved by the Huelva Regional Research Ethics Committee (2534‐N‐21). Patients who were not assessed through preoperative MRI were excluded from the study.

Patients' medical histories were studied in order to detect risk factors for lymph node metastasis. The variables used were: (1) histological type, (2) tumor grade, (3) myometrial invasion, (4) Ca125 level, (5) MRI volume index, and (6) lymphovascular space invasion (LVSI).

Every variable was stratified using a binary classification. (1) Histological type was classified into endometroid/nonendometroid, (2) tumor grade into G1/G2 versus G3. (3) Myoinvasion was classified according to the revised FIGO system 50% versus ≥50%. (4–5) Volume index and Ca125 level serum were classified into two categories using the measurement obtained by the Receiver Operating Characteristics (ROC) curve for lymph node metastasis based on sensitivity and specificity. LVSI determination was reported preoperatively and confirmed on the final histology in all included patients.

### 2.2 Imaging protocol

Preoperative pelvic MRI was performed using equipment with a 1.5 T magnetic field (Phillips Healthcare or General Electric Medical Systems). High‐resolution T2‐weighted turbo spin‐echo sequences were performed (3 mm slice thickness) in the three orthogonal or oblique planes appropriate for the major and minor axes of the body of the uterus, as well as diffusion‐weighted planar echo sequence (DWI) with a maximum b value >600 s/mm^2^ and corresponding apparent diffusion coefficient (ADC) maps. Additional sequences (weighted on T1 or T2 with fat saturation) as well as the use of T1 sequences with fat saturation after administration of gadolinium were used at the discretion of the supervising radiologist of the study. MRIs were analyzed by a radiologist with more than 10 years of experience in gynecological cancer imaging.

The estimated volumes of the neoplasm and uterus have been calculated using an ellipsoid formula (Longitudinal ×  Transverse × Anteroposterior × [*π*/3]). The neoplasm/uterus volume ratio (N/U) was calculated. Subsequently, a manual calculation of the volume was carried out by contouring the lesions or region of interest (ROI) in which the neoplasm showed the largest size in T2W, using the standard software (LiveWire, Carestream, or Phillips VUE PACS tool).

### 2.3 Statistical analysis

Continuous variables are reported as mean ± standard deviation (SD) or median with interquartile range (IQR), and all categorical variables are reported as number and percentage. Normality was evaluated using the Kolmogorov–Smirnov test. Comparisons between categorical variables were analyzed using the Chi‐squared test. For continuous variables, comparisons were made using the Mann–Whitney *U* test or Kruskal–Wallis, as appropriate. For multiple comparisons, the Bonferroni correction was applied. Longitudinal comparisons were made through the Wilcoxon test. Analyses of ROC were performed to assess the optimal cut‐off value and diagnostic accuracy of lymph node metastases. To estimate the diagnostic accuracy agreement between the optimal cut‐off points in the different tumor volume parameters, Cohen's kappa statistic was used, so that *κ* = 0.01–0.20 (slight), *κ* = 0.21–0.40 (fair), *κ* = 0.41–0.60 (moderate), *κ* = 0.61–0.80 (substantial), and *κ* ≥ 0.81 (almost perfect). To study the predictive factors that determine lymph node metastases as independent risk factors, a logistic regression analysis was performed. Initially, a univariate analysis was performed and those factors with a significance below *p* = 0.1 were the variables included in the multivariate logistic regression analysis as independent, thus establishing the regression model. Odds Ratio (OR) and Confidence Intervals (CI) were calculated and reported. In all cases, a statistical significance of 5% (*p* < 0.05) was required. Statistical analyses were performed using the SPSS statistical program, version 24.0.

## 3 RESULTS

A total of 194 patients with endometrial cancer were identified over the period of study with a mean follow‐up period of 66 months.

Surgical and baseline characteristics were as follows: the median age was of 63.73 years (range 28–91) and 82.56% were postmenopausal. The predominant histological subtype was endometrioid with a rate of 84.5%. Of the 194 patients in the cohort, 179 underwent complete surgical staging and ninety‐seven patients (54.18%) had pelvic lymph node sampling, while 43 (24.02%) had accompanying para‐aortic lymph node sampling. The mean number of nodes dissected was 13 (range 4–24) in the pelvic lymphadenectomy and 20 (range 5–30) in the para‐aortic lymphadenectomy. Regarding the stage of the patients, 80 were IA FIGO stage (41%), while 50 were FIGO stage IB (25.3%); 1 (0.5%) had cervical stromal invasion (FIGO stage II); 43 were stage III (22.1%); and 7 were stage IV (3.6%). 20 patients (12.2%) presented lymph node metastases. Adjuvant treatment was given to 117 (60.30%) patients; 46 (23.71%) were submitted to chemotherapy; external radiation therapy was applied in 10 patients (5.1%); brachytherapy was administered in 98 (50.51%) of the patients; and 6 (3%) patients were treated with hormonal treatment (Table 1).

**TABLE 1:** Characteristics of endometrial cancer patients (*N* = 194).

Characteristics	Median (range, %)
Age median (range)	63.73 years (28–91)
Postmenopausal status *N* (%)	161 (82.56%)
Histopathologic subtypes *N* (%)	
Endometrioid	164 (84.5%)
Nonendometroid	30(15.5%)
Surgical lymphadenectomy	
Pelvic	97 (54%)
Pelvic + aortic	43 (24.02%)
Mean number lymphadenectomy	
Pelvic	13 (4–24)
Aortic	20 (5–30)
FIGO stage	
I	130 (67%)
II	1 (0.5%)
III	43 (22%)
IV	7 (3.6%)
Adjuvant treatment	117 (60.30%)
Chemotherapy	46 (23.71%)
External radiation therapy	10 (5.1%)
Brachytherapy	98 (50.51%)
Hormonal treatment	6 (3%)

**FIGURE 1:**
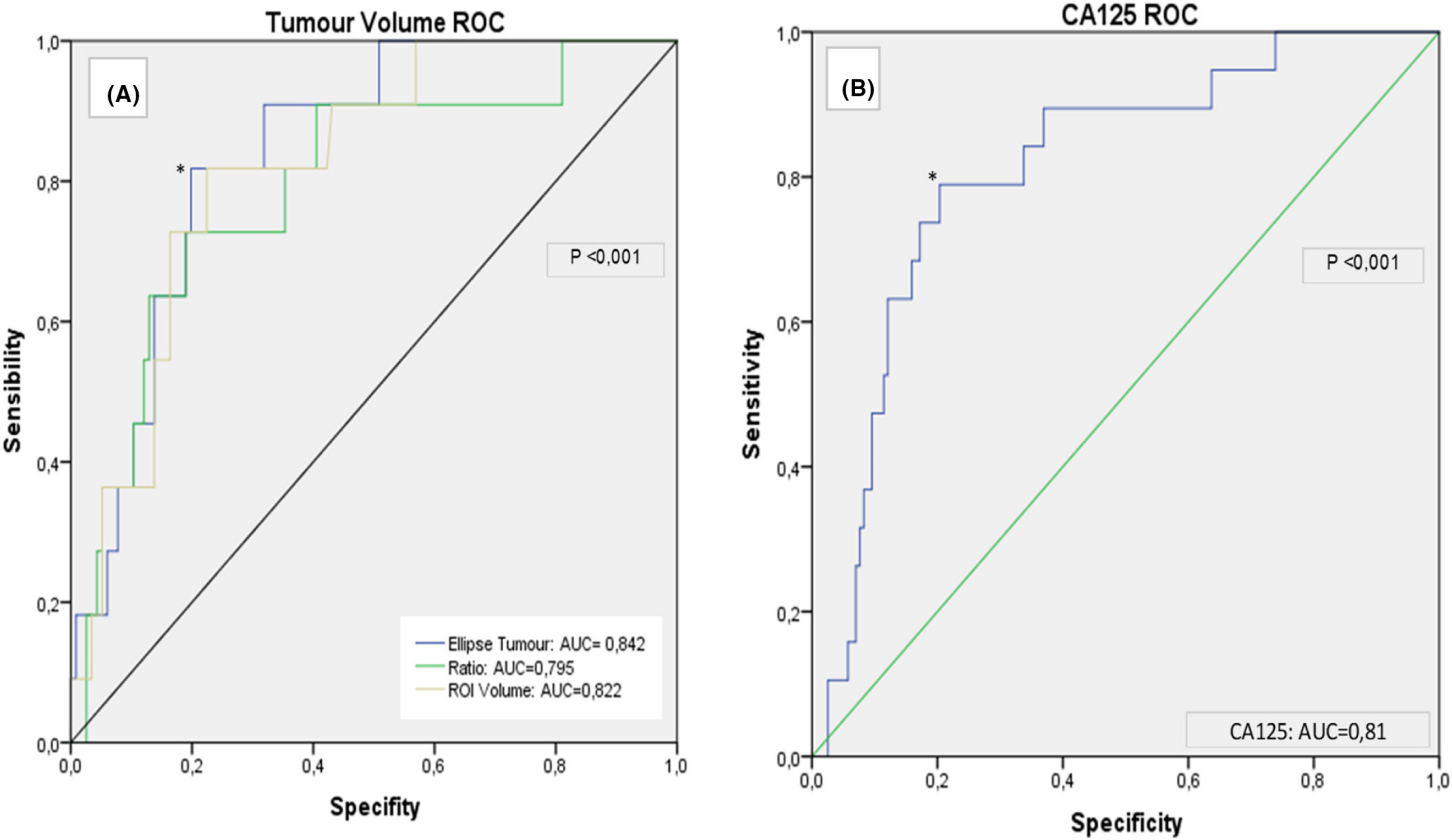
(A) Receiver operator characteristic (ROC) curves for the various tumor size measurements for identification of lymph node metastases and (B) ROC curves for Ca125 level to identify node metastases.

To study different risk factors, the analysis detected different ROC curves for the Tumor Volume parameters with the Tumor Ellipse, Ratio, and ROI, obtaining a cut‐off value to be applied in the prediction of lymph node metastases through these different approaches. A cut‐off value ≥25 cm^3^ tumor volume was identified as a predictor of lymph node metastases with a sensitivity and specificity of S72% and E84% and area under the curve (AUC) of 0.82. A cut‐off value of 28 of CA 125 classified node lymph metastasis into two groups of high and low risk, through ROC curves with an AUC of 0.81 and sensitivity and specificity of S78% and E80% (Figure 1).

Univariate analysis revealed as risk factors significantly associated with node metastases the Histological grade (Grade 3) (*p* = 0.001), volume index >25 (*p* < 0.001), LVSI (*p* = 0.002), and Ca 125 (>28) (*p* < 0.001). There was no significant difference in MRI between patients of age > 60 (*p* = 0.85) and myometrial invasion ≥50% (*p* = 0.20). Multivariate analysis confirmed that volume index >25 (odd ratio [OR] 0.64, 95% confidence interval [CI] = 0.09–1.046), Ca 125 > 28 (OR = 0.32; 95% CI 0.03–1.32), and high histological grade (OR = 2.6, 95% CI = 1.1–3.2) were independent prognostic factors to lymph node metastasis. LVSI (*p* = 0.4) was not a significant independent risk factor for lymph node metastases, therefore, it was not included in the multivariate study (Table 2).

**TABLE 2:** Univariate and multivariate analysis of factors for lymph node metastasis.

	Node metastases		Univariate analysis	Multivariate analysis	
Variable	No	Yes	*p* value	OR	*p* value
LVSI invasion					
Negative	91	4	0.002		0.104
Positive	25	7			
Age					
<60 years	39	4	0.854		0.9
>60 years	77	7			
Ca125 level					
Low <28.48	97	2	<0.001	0.32	0.004
High ≥28.48	19	9			
Volume index					
<25.45	93	2	<0.001	0.64	0.007
≥25.45	23	9			
Grade					
G1/G2	100	6	0.007	2.664	0.014
G3	16	5			
Myometrial invasion MRI					
<50%	55	3	0.2		0.817
≥50%	61	8			

Based on the analysis of the results, an algorithm was created according to the relationship between the related preoperative significant risk factors (LVSI, G3, Volume Index >25 and CA125 > 28) and the presence of lymph node metastasis. The algorithm stratified patients according to the presence of risk factors. Patients with no significant risk factors did not present lymph node metastases. In cases with one risk factor (Ca125 > 28), the rate was 1.3%, which rose to 20%, 40%, and 80% in cases with two, three, and more than three risk factors, respectively.

**FIGURE 2:**
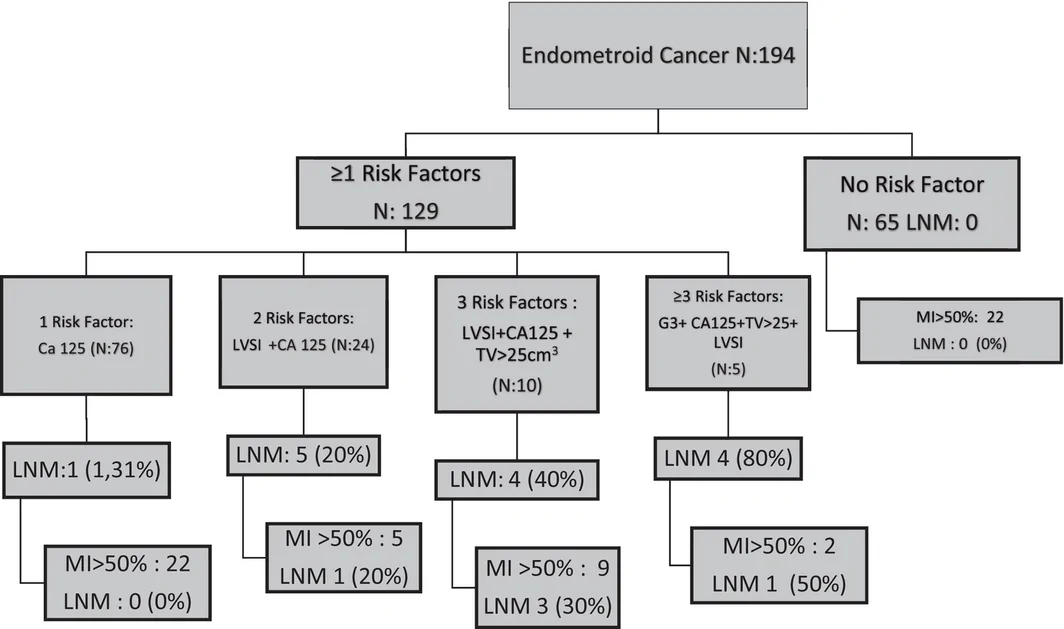
Patients and rate of lymph node metastases (LNM) according to the number of risk factors Number of patients and rate of lymph node metastasis (LNM) according to the number of risk factors. CA125 > 28, lymphovascular space invasion (LVSI), tumor volume (TV) > 25 cm, high‐grade tumoral (G3). Adjusted by myometrial invasion >50% (MI > 50%).

Patients with a myometrial invasion ≥50% were then assessed. The algorithm was adjusted with patients with a deep myometrial invasion ≥50%. This risk factor was not significant in the analysis, but it was used to assess the tailored surgery in daily practice. When included, it was observed that its presence did not negatively influence subsequent results. In the population without risk factors (*N* = 65), 22 patients presented deep myometrial invasion, all of them with no node metastases associated. In the group with only one risk factor (Ca 125), the presence of myometrial invasion was found in only 22 patients, and the presence of lymph node metastases disappeared. In cases with two risk factors, the adjusted rate of lymph node metastases was 20%, which rose to 30% and 50% in the group with three or more than three, respectively (Figure 2).

## 4 DISCUSION

Tumor size is a prognosis factor that determines the postsurgery risk classification in EC. According to the Oncology Guide of the Spanish Society of Gynecology and Obstetrics, recently updated in 2023, a size >2 cm in the final surgical specimen, together with age > 60 years, high‐grade tumor (G3), and extensive LVSI involvement, are poor prognostic criteria that suggest the use of postsurgical adjuvant therapy.^18^ However, tumor size is not included in the stage and pre‐surgery assessment, as in other gynecology tumors, so it is not a determining factor when deciding upon performing lymphadenectomy. The present study shows the introduction of the size of tumor based on the volume as a new risk factor for predicting the nodal status in EC. Of the 65 cases without the established risk factors for affected lymph node, metastasis was found in 0 cases. None of the patients who lacked the established risk factors criteria had lymph node metastasis.

Lymph node involvement is an independent prognostic factor,^4^ and this has already been shown in the previous analysis with an OR = 3.405 associated with recurrence. The risk of lymph node metastasis in low‐risk patients is 3%–5%, while it rises to 20% in high‐risk patients.^6^, ^19^, ^20^ The Mayo Clinic criteria is widely accepted to assess the level of risk of lymph node metastases. Low risk includes endometroid/grade 1–2 histological type, 50% or less myometrial invasion depth, and tumor diameter of 2 cm or less.^6^ It is known that the presence of lymph node metastasis is essential to plan adjuvant treatment. Lymphadenectomy is indicated in intermediate‐high‐risk patients, as supported by studies.^21^, ^22^, ^23^ However, patients without nodal involvement do not obtain any benefit, resulting in increased comorbidity.^24^, ^25^ Therefore, an accurate and tailored presurgical risk assessment is needed in order to decide whether or not to perform a lymphadenectomy.

In fact, several studies have previously proposed the criterion for assessing the risk of EC. Mariani^6^ used the Mayo criteria to select 328 low‐risk patients to omit lymphadenectomy, finding a recurrence‐free and overall survival at 5 years of 96% and 97%, respectively. However, these criteria were adjusted to the postsurgical findings. Another low‐risk criterion for the omission was proposed based on preoperative tests, but the imaging of myometrial invasion and lymph node involvement had a misdiagnosis rate of up to 20% and did not assess the tumor size on imaging.^26^ The classic criteria were also analyzed in several studies: regarding the depth of myometrial invasion with MRI,^6^, ^26^ there were misleading cases, and concerning the histological grade of the tumor, there was a difference between the grade determined in the previous biopsy and the hysterectomy specimen.^27^

The discrepancy between the pre‐ and postsurgery findings generated the search for new preoperative tools that allowed a correct tailored surgery. Preoperative tumor volume cannot be routinely correlated with definitive histology, but it is a radiological parameter independent of myometrial infiltration. The introduction of ROI as a method of volume measurement is especially useful with tumors given their irregular contour, which does not allow for other traditional calculations such as the ellipse formula. Some authors have recently introduced tumor size in the algorithm through PET imaging,^28^ but it would require an additional imaging technique, not available in all centers. Finally, there are studies that have introduced volume as an independent prognostic factor for lymph node metastases,^28^, ^29^ but they have not succeeded in achieving a null rate of lymph node involvement that would allow avoiding the lymphadenectomy safely, and only one of these studies^30^ proposed an own cut‐off point. As for the serum tumor markers, elevation of Ca 125 was significantly associated with poor prognosis in the previous analysis, consistent with what is described in the literature.^31^, ^32^

Limitations of the study include omitting molecular parameters, recently introduced in the algorithm for the management of EC, as well as omitting the sentinel lymph node since it was not studied in the entire study population and is a source of bias. Also, the inability to correlate the preoperative radiologic tumor volume with the volume of the surgical specimen is considered a limitation.

Based on all the above described, the volume index as a new MRI risk factor was introduced in the present study, finding an optimal cut‐off point (<25 cm^3^) associated with a null rate of lymph node metastases. Myometrial invasion on MRI was not a significant risk factor for node metastasis in the performed analysis, so it was omitted as a risk factor in the strategy. Based on the findings, high‐grade tumor, Ca 125 elevated (>28), and Tumor volume on MRI greater than 25 cm^3^ were considered high‐risk factors for node metastasis.

The null rate of node metastasis when the selected risk factors were not present and the low interparameter variability are the main differences with the published literature published to date. Given these findings, a set of criteria that include three elements is proposed: tumor volume < 25 cm^3^; CA 125 < 28 UI/mL; and preoperative tumor grade 1 or 2. The results of the present study allow applying this proposed algorithm to omit lymphadenectomy on the basis of safe criteria.

## 5 CONCLUSION

The findings of the present study proved, among other facts, that the multivariate analysis confirmed that volume index >25 (OR = 0.64, 95% CI = 0.09–1.046), Ca 125 > 28 (OR = 0.32; 95% CI 0.03–1.32), and high histological grade (OR = 2.6, 95% CI = 1.1–3.2) were independent prognostic factors for lymph node metastasis.

To sum up, the proposed algorithm score may provide useful information for stratification of the risk of node metastasis, and the present findings suggest that lymphadenectomy can be omitted in cases with no risk factors, according to this node metastasis score.

## AUTHOR CONTRIBUTIONS

**Elga López‐González:** Conceptualization (equal); data curation (equal); formal analysis (equal); investigation (equal); methodology (equal); resources (equal); software (equal); supervision (equal); validation (equal); visualization (equal); writing – original draft (equal); writing – review and editing (equal). **Alberto Rodríguez‐Jiménez:** Conceptualization (equal); data curation (equal); formal analysis (equal); investigation (equal); methodology (equal); resources (equal); software (equal); validation (equal); visualization (equal); writing – original draft (equal); writing – review and editing (equal). **José Antonio Rojas‐Luna:** Conceptualization (equal); data curation (equal); formal analysis (equal); investigation (equal); methodology (equal); resources (equal); supervision (equal); validation (equal); visualization (equal); writing – original draft (equal); writing – review and editing (equal). **Cinta Daza‐Manzano:** Conceptualization (equal); data curation (equal); formal analysis (equal); investigation (equal); methodology (equal); resources (equal); software (equal); validation (equal); visualization (equal); writing – original draft (equal); writing – review and editing (equal). **Juan Gómez‐Salgado:** Conceptualization (equal); data curation (equal); formal analysis (equal); investigation (equal); methodology (equal); project administration (equal); resources (equal); software (equal); supervision (equal); validation (equal); visualization (equal); writing – original draft (equal); writing – review and editing (equal).

## FUNDING INFORMATION

There were no sources of funding.

## CONFLICT OF INTEREST STATEMENT

The authors declare that they have no competing interests.

## Data Availability

All data are available within this article.

## References

[1] Sung H , Ferlay J , Siegel RL , et al. Global Cancer Statistics 2020: GLOBOCAN estimates of incidence and mortality worldwide for 36 cancers in 185 countries. CA Cancer J Clin. 2021;71(3):209‐249. doi:10.3322/caac.21660 33538338

[2] Siegel RL , Miller KD , Jemal A . Cancer statistics, 2020. CA Cancer J Clin. 2020;70(1):7‐30. doi:10.3322/caac.21590 31912902

[3] Gitas G , Freytag D , Allahqoli L , et al. Lymphadenectomy in endometrial cancer—achieving more with less? Minim Invasive Ther Allied Technol. 2022;31(4):531‐539. doi:10.1080/13645706.2020.1868009 33439061

[4] Swift BE , Philp L , Atenafu EG , Malkani N , Gien LT , Bernardini MQ . Lymphadenectomy for high‐grade endometrial cancer: does it impact lymph node recurrence? Eur J Surg Oncol. 2022;48(5):1181‐1187. doi:10.1016/j.ejso.2021.11.009 34782183

[5] Fasmer KE , Hodneland E , Dybvik JA , et al. Whole‐volume tumor MRI radiomics for prognostic modeling in endometrial cancer. J Magn Reson Imaging. 2021;53(3):928‐937. doi:10.1002/jmri.27444 33200420 PMC7894560

[6] Mariani A , Webb MJ , Keeney GL , Haddock MG , Calori G , Podratz KC . Low‐risk corpus cancer: is lymphadenectomy or radiotherapy necessary? Am J ObstetGynecol. 2000;182(6):1506‐1519. doi:10.1067/mob.2000.107335 10871473

[7] Concin N , Matias‐Guiu X , Vergote I , et al. ESGO/ESTRO/ESP guidelines for the management of patients with endometrial carcinoma. Int J Gynecol Cancer. 2021;31(1):12‐39. doi:10.1136/ijgc-2020-002230 33397713

[8] Frei KA , Kinkel K . Staging endometrial cancer: role of magnetic resonance imaging. J Magn Reson Imaging. 2001;13:850‐855. 11382943 10.1002/jmri.1121

[9] Kinkel K , Forstner R , Danza FM , et al. Staging of endometrial cancer with MRI: guidelines of the European Society of Urogenital Imaging. Eur Radiol. 2009;19:1565‐1574. 19194709 10.1007/s00330-009-1309-6

[10] Sala E , Rockall AG , Freeman SJ , Mitchell DG , Reinhold C . The added role of MR imaging in treatment stratification of patients with gynecologic malignancies: what the radiologist needs to know. Radiology. 2013;266(3):717‐740. doi:10.1148/radiol.12120315 23431227

[11] Alcazar JL , Pineda L , Martinez‐Astorquiza Corral T , et al. Transvaginal/transrectal ultrasound for assessing myometrial invasion in endometrial cancer: a comparison of six different approaches. J Gynecol Oncol. 2015;26:201‐207. 26197857 10.3802/jgo.2015.26.3.201 PMC4510336

[12] Haldorsen IS , Salvesen HB . Staging of endometrial carcinomas with MRI using traditional and novel MRI techniques. Clin Radiol. 2012;67:2‐12. 22119292 10.1016/j.crad.2011.02.018

[13] Todo Y , Choi HJ , Kang S , et al. Clinical significance of tumor volume in endometrial cancer: a Japan–Korea cooperative study. Gynecol Oncol. 2013;131(2):294‐298. doi:10.1016/j.ygyno.2013.08.008 23954595

[14] Todo Y , Watari H , Okamoto K , et al. Tumor volume successively reflects the state of disease progression in endometrial cancer. Gynecol Oncol. 2013;129(3):472‐477. doi:10.1016/j.ygyno.2013.02.034 23474346

[15] Mahdi H , Munkarah AR , Ali‐Fehmi R , Woessner J , Shah SN , Moslemi‐Kebria M . Tumor size is an independent predictor of lymph node metastasis and survival in early stage endometrioid endometrial cancer. Arch Gynecol Obstet. 2015;292(1):183‐190. doi:10.1007/s00404-014-3609-6 25549769

[16] Canlorbe G , Bendifallah S , Laas E , et al. Tumor size, an additional prognostic factor to include in low‐risk endometrial cancer: results of a French multicenter study. Ann Surg Oncol. 2016;23(1):171‐177. doi:10.1245/s10434-015-4583-3 25952272

[17] Chattopadhyay S , Cross P , Nayar A , Galaal K , Naik R . Tumor size: a better independent predictor of distant failure and death than depth of myometrial invasion in international federation of gynecology and obstetrics stage I endometrioid endometrial cancer. Int J Gynecol Cancer. 2013;23(4):690‐697. doi:10.1097/igc.0b013e31828c85c6 23518862

[18] Sociedad Española de Ginecología y Obstetricia . Oncoguía SEGO: Cáncer de endometrio. SEGO; 2023. ISBN: 978‐84‐09‐40278‐6.

[19] Mariani A , Dowdy SC , Cliby WA , et al. Prospective assessment of lymphatic dissemination in endometrial cancer: a paradigm shift in surgical staging. Gynecol Oncol. 2008;109(1):11‐18. doi:10.1016/j.ygyno.2008.01.023 18304622 PMC3667391

[20] Turan T , Hizli D , Sarici S , et al. Is it possible to predict Para‐aortic lymph node metastasis in endometrial cancer? Eur J Obstet Gynecol Reprod Biol. 2011;158(2):274‐279. doi:10.1016/j.ejogrb.2011.04.031 21664758

[21] Todo Y , Kato H , Kaneuchi M , Watari H , Takeda M , Sakuragi N . Survival effect of para‐aortic lymphadenectomy in endometrial cancer (SEPAL study): a retrospective cohort analysis. Lancet. 2010;375(9721):1165‐1172. doi:10.1016/S0140-6736(09)62002-X 20188410

[22] Katz LA , Andrews SJ , Fanning J . Survival after multimodality treatment for stage IIIC endometrial cancer. Am J Obstet Gynecol. 2001;184(6):1071‐1073. doi:10.1067/mob.2001.115225 11349160

[23] Bristow RE , Zahurak ML , Alexander CJ , Zellars RC , Montz FJ . FIGO stage IIIC endometrial carcinoma: resection of macroscopic nodal disease and other determinants of survival. Int J Gynecol Cancer. 2003;13(5):664‐672. doi:10.1136/ijgc-00009577-200309000-00015 14675352

[24] Uccella S , Podratz KC , Aletti GD , Mariani A . Re: systematic pelvic lymphadenectomy vs no lymphadenectomy in early‐stage endometrial carcinoma: randomized clinical trial. J Natl Cancer Inst. 2009;101(12):897‐898; Author reply 898‐899. doi:10.1093/jnci/djp124 19509367

[25] Benedetti Panici P , Basile S , Maneschi F , et al. Systematic pelvic lymphadenectomy vs. no lymphadenectomy in early‐stage endometrial carcinoma: randomized clinical trial. J Natl Cancer Inst. 2008;100(23):1707‐1716. doi:10.1093/jnci/djn397 19033573

[26] Kim M , Choi C , Kim K , et al. Three‐year recurrence‐free survival in patients with a very low risk of endometrial cancer who did not undergo lymph node dissection (tree retro): a Korean multicenter study. Int J Gynecol Cancer. 2018;28(6):1123‐1129. doi:10.1097/IGC.0000000000001270 29664841

[27] Obermair A , Geramou M , Gücer F , et al. Endometrial cancer: accuracy of the finding of a well differentiated tumor at dilatation and curettage compared to the findings at subsequent hysterectomy. Int J Gynecol Cancer. 1999;9(5):383‐386. doi:10.1046/j.1525-1438.1999.99050.x 11240798

[28] Zhang G , Chen H , Liu Y , et al. Is lymph node dissection mandatory among early stage endometrial cancer patients? A retrospective study. BMC Womens Health. 2020;20(1):258. doi:10.1186/s12905-020-01128-w 33213444 PMC7678324

[29] Todo Y , Sakuragi N , Nishida R , et al. Combined use of magnetic resonance imaging, CA 125 assay, histologic type, and histologic grade in the prediction of lymph node metastasis in endometrial carcinoma [published correction appears in Am J Obstet Gynecol. 2003 Aug;189(2):567]. Am J Obstet Gynecol. 2003;188(5):1265‐1272. doi:10.1067/mob.2003.318 12748496

[30] Matsushita C , Fujiwara H , Takei Y , et al. New criteria for the omission of lymphadenectomy in endometrioid carcinoma. Int J Gynecol Cancer. 2019;29(3):541‐546. doi:10.1136/ijgc-2018-000044 30630888

[31] Takeshima N , Shimizu Y , Umezawa S , et al. Combined assay of serum levels of CA125 and CA19‐9 in endometrial carcinoma. Gynecol Oncol. 1994;54(3):321‐326. doi:10.1006/gyno.1994.1217 8088608

[32] Sood AK , Buller RE , Burger RA , Dawson JD , Sorosky JI , Berman M . Value of preoperative CA125 level in the management of uterine cancer and prediction of clinical outcome. Obstet Gynecol. 1997;90:441‐447. 9277659 10.1016/s0029-7844(97)00286-x

